# Tolerogenic effects of 1,25-dihydroxyvitamin D on dendritic cells involve induction of fatty acid synthesis

**DOI:** 10.1016/j.jsbmb.2021.105891

**Published:** 2021-07

**Authors:** Amadeo Muñoz Garcia, Emma L. Bishop, Danyang Li, Louisa E. Jeffery, Antje Garten, Alpesh Thakker, Michelangelo Certo, Claudio Mauro, Daniel A. Tennant, Sarah Dimeloe, Chris T. Evelo, Susan L. Coort, Martin Hewison

**Affiliations:** a Institute of Metabolism and Systems Research, University of Birmingham, Birmingham, B15 2TT, United Kingdom; b Department of Bioinformatics-BiGCaT, NUTRIM School of Nutrition and Translational Research in Metabolism, Maastricht University, Maastricht, Netherlands; c Institute of Immunology and Immunotherapy, University of Birmingham, Birmingham, B15 2TT, United Kingdom; d Institute of Translational Medicine, University of Birmingham, Birmingham, B15 2TT, United Kingdom; e Universität Leipzig, Medizinische Fakultät, Leipzig, 04103, Germany; f Institute of Inflammation and Ageing, University of Birmingham, Birmingham, B15 2TT, United Kingdom; g Maastricht Centre for System Biology (MaCSBio), Maastricht University, Maastricht, Netherlands

**Keywords:** Vitamin D, Dendritic cell, Metabolism, Glycolysis, TCA cycle, Fatty acid synthesis

## Abstract

The active form of vitamin D, 1,25-dihydroxyvitamin D (1,25D) is a potent regulator of immune function, promoting anti-inflammatory, tolerogenic T cell responses by modulating antigen presentation by dendritic cells (DC). Transcriptomic analyses indicate that DC responses to 1,25D involve changes in glycolysis, oxidative phosphorylation, electron transport and the TCA cycle. To determine the functional impact of 1,25D-mediated metabolic remodelling, human monocyte-derived DC were differentiated to immature (+vehicle, iDC), mature (+LPS, mDC), and immature tolerogenic DC (+1,25D, itolDC) and characterised for metabolic function. In contrast to mDC which showed no change in respiration, itolDC showed increased basal and ATP-linked respiration relative to iDC. Tracer metabolite analyses using ^13^C -labeled glucose showed increased lactate and TCA cycle metabolites. Analysis of lipophilic metabolites of ^13^C-glucose revealed significant incorporation of label in palmitate and palmitoleate, indicating that 1,25D promotes metabolic fatty acid synthesis in itolDC. Inhibition of fatty acid synthesis in itolDC altered itolDC morphology and suppressed expression of CD14 and IL-10 by these cells. These data indicate that the ability of 1,25D to induce tolerogenic DC involves metabolic remodelling leading to synthesis of fatty acids.

## Highlights

• Dendritic cells (DC) treated with 1,25-dihydroxyvitamin D (1,25D) alone show a tolerogenic DC phenotype.
• 1,25D-treated DC show altered oxidative phosphorylation and electron transport.
• 1,25D-treated DC show altered glycolysis and TCA cycle metabolism.
• TCA effects of 1,25D enhance fatty acid synthesis by DC.
• Fatty acid synthesis appears to be essential for DC tolerogenic effects of 1,25D.

## Graphical abstract

**Figure:**
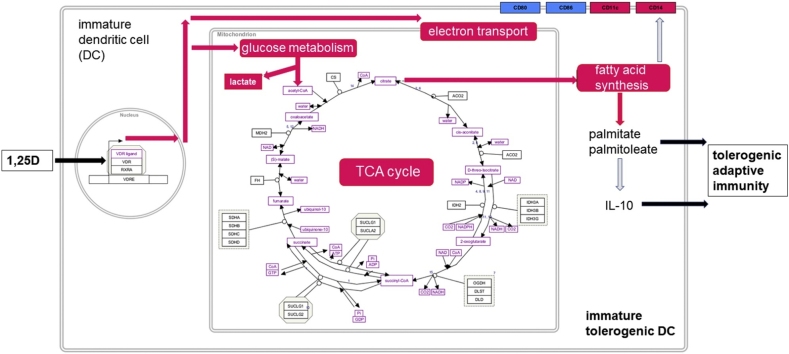

## 1 Introduction

The active form of vitamin D, 1,25-dihydroxyvitamin D (1,25D), is a potent immunomodulator, promoting innate antibacterial activity [1,2], whilst inhibiting inflammatory acquired immune responses [2,3]. Similar to its classical calciotropic actions, the immunomodulatory activities of 1,25D are mediated by binding to the nuclear vitamin D receptor (VDR) [4], and concomitant regulation of transcription [5]. Whilst VDR expression is ubiquitous, the level of VDR is known to vary significantly in cells from the immune system. Resting T cells exhibit very low levels of VDR, with expression increasing dramatically following exposure to immunogens and T cell activation [6]. In this way 1,25D is able to regulate activated T cells directly by supressing T helper 1 (Th1) and Th17 function and promoting differentiation into regulatory T cells (Treg) [7]. However, 1,25D can also influence adaptive immunity T cell function via indirect effects on antigen presenting cells. Macrophages [8] and dendritic cells (DC) [9] express VDR, suggesting that regulation of antigen presentation to T cells may be a key function of 1,25D.

The mechanisms by which 1,25D modulates DC function have still to be fully elucidated, and are likely to be dependent on specific DC phenotype, and the immune stimulus for the DC. However, these monocyte-derived immature DC (iDC) can be further modulated to generate DC with specific antigen-presenting cell characteristics. Activation of iDC with immune modulators such as lipopolysaccharide (LPS) generates mature DC (mDC), with enhanced expression of cell surface antigens such as CD80 and CD86, co-stimulatory molecules required for antigen-presentation to T-cells [10]. Treatment of mDC with 1,25D suppresses expression of CD80/CD86 [9], and cytokines such as IL-12 associated with T cell activation [9]. However, 1,25D also increases DC expression of other cytokines such as IL-10 [11], to limit the maturation of DC [9,12], and promote a tolerogenic DC phenotype (tolDC) [13,14] which can elicit development of Treg [15].

To date studies of 1,25D responses in DC have focused on LPS-induced mDC, with the resulting cells being characterised as mature tolerogenic DC (mtolDC) [16,17]. Much less is known about DC effects of 1,25D in the absence of immune activation. Studies by our group and others have demonstrated expression of VDR in both iDC and mDC [9,18], indicating that both types of DC are potential targets for 1,25D. In murine bone marrow-derived DC, 1,25D was shown to regulate a greater number of genes in LPS-induced mtolDC relative to DC treated with 1,25D alone (referred to in the current study as immature tolerogenic DC (itolDC)) [19]. Whilst some 1,25D-regulated genes were common to both cell types, many were specific to mtolDC or itolDC [19]. Thus, the maturation context of DC responses to 1,25D is likely to be critical in defining the impact of vitamin D on innate immune DC function.

Recent reports have shown that the effects of 1,25D on mDC are crucially dependent on metabolic remodelling. In monocyte-derived, LPS-induced mDC, pathway analyses showed that the dominant transcriptomic effects of 1,25D involved changes in glucose metabolism, oxidative phosphorylation and the TCA cycle [20]. Specifically, short-term treatment of LPS-induced mDC with 1,25D induced an mtolDC phenotype that was dependent on glucose availability and glycolysis [20]. In studies using multiple gene expression repositories we have shown that the ability of 1,25D to promote metabolic remodelling via glycolysis, oxidative phosphorylation and the TCA cycle is common to many myeloid cell types including monocytes, iDC and mDC [21]. In the current study we have further defined the role of metabolic remodelling in mediating immunomodulatory effects of 1,25D by demonstrating increased fatty acid synthesis by DC in the absence of immunogenic stimulus. Inhibition of fatty acid synthase suppressed the ability of 1,25D to promote IL-10-secreting itolDC, indicating that regulation of lipid metabolism is central to the immunomodulatory actions of 1,25D.

## 2 Materials & methods

### 2.1 Isolation of primary human peripheral blood monocytes

Healthy human peripheral blood mononuclear cells (PBMC) were obtained from fully anonymised blood cones obtained from the National Blood Service, Birmingham, UK, in accordance with ethical agreement ERN_14-0446. PBMC were isolated from whole blood leukocyte cones from healthy donors using LymphoPrep Separation Media (Stem Cell Technologies, Cambridge, UK) as per manufacturer’s instructions, and resuspended in ice-cold MACS (magnetic-activated cell sorting) buffer to achieve approximately 50million PBMCs per mL. CD14+ monocytes were isolated from PBMC using the EasySep Monocyte Isolation Kit (Stem Cell Technologies) as per manufacturer’s instructions. The yield of CD14+ monocytes achieved from 500 million PBMC was approximately 50 million. The resulting monocytes were transferred to a new tube, and PBS added up to 15 mL. Following centrifugation at 1500 rpm for 5 min, pelleted CD14+ monocytes were re-suspended in Roswell Park Memorial Institute (RPMI) 1640 Medium (Thermofisher, Loughborough, UK) supplemented with 5% l-glutamine (Sigma Aldrich, Gillingham, UK) and 10 % foetal bovine serum (FBS; Biosera, Heathfield, UK) to achieve 2 million cells per mL.

### 2.2 In vitro generation of monocyte-derived DC

**Fig. 1:**
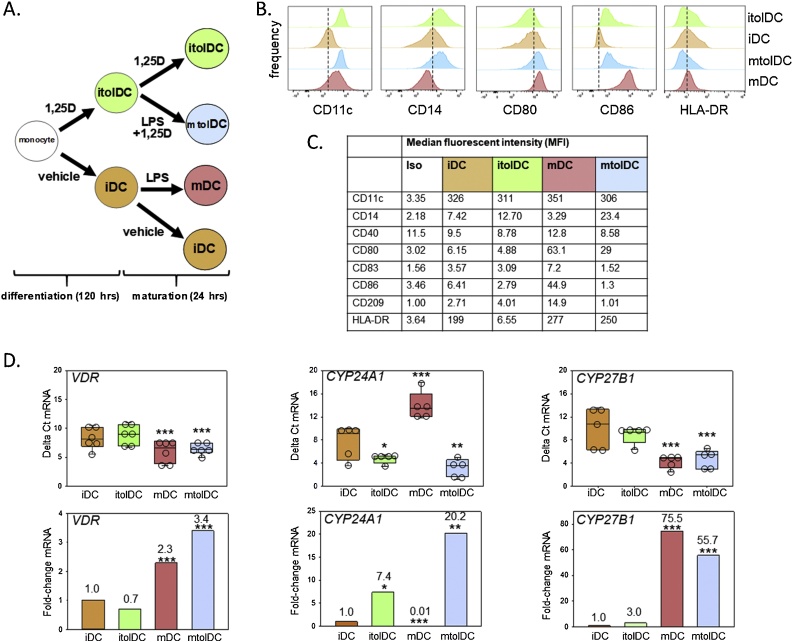
**Regulation of DC phenotype by 1,25D.** A. Schematic representation of the model for cell culture of human monocyte-derived DC. Immature DC (iDC), mature DC (mDC), immature tolerogenic DC (itolDC), mature tolerogenic DC (mtolDC). B. Representative flow cytometry analyses for CD11c, CD14, CD80, CD86 and HLA-DR in iDC, mDC, mtolDC and itolDC showing comparison between iDC vs itolDC, iDC vs mDC and mDC vs mtolDC. C. Median fluorescence intensity values for CD11c, CD14, CD40, CD80, CD83, CD86, CD209 and HLA-DR in iDC, mDC, mtolDC and itolDC. D. Expression of mRNA for *VDR*, *CYP24A1* and *CYP27B1* in iDC, mDC, mtolDC and itolDC. Data are shown as: raw delta Ct values with associated statistical analysis (upper panel); fold-change in mRNA expression (lower panel). Data show individual replicate cell preparation values and median and upper/lower quartiles for n = 5 replicate donor analyses. * = statistically different from iDC values, p < 0.05, ** p < 0.01, *** p < 0.001.

Individual donor monocyte preparations outlined above were used to generate the different DC phenotypes for each experiment. Each donor therefore acted as a replicate for each experiment, with associated intra-donor variability. For each donor, CD14+ monocytes were differentiated into DC in vitro according to the schematic shown in Fig. 1A. Monocytes were cultured for 5 days with granulocyte macrophage-stimulating colony factor (GM-CSF; 800U/mL, Berlex Laboratories, Seattle, WA) and Interleukin-4 (IL-4; 400U/mL), in the presence or absence of 10 nM 1,25D (Enzo Life Sciences, Exeter, UK; diluted in RPMI 1640 medium from 50 μg/mL stock), at 37 °C and 5% CO₂. Fresh medium supplemented with GM-CSF and IL-4 was added on day 2 and day 5 of culture, with the resulting day 6 cells being immature DC (iDC). The addition of 1 μg/mL LPS (from *E. coli*, Sigma Aldrich) for 24 h on day 6 generated mature DC (mDC). Addition of 1,25D (10 nM) for all 6 days of culture in the absence of LPS was used to generate immature tolerogenic DC (itolDC) and the addition of LPS for the last 24 h of these cultures generated mature tolerogenic DC (mtolDC). In some cultures mitochondrial fatty acid synthase (FAS) was specifically inhibited using 25 μM of 4-Methylene-2-octyl-5-oxotetrahydrofuran-3-carboxylic acid (C75, Sigma Aldrich) from day 0.

### 2.3 Flow cytometry analysis of DC

Approximately 50,000 cells were transferred directly from culture plates into flow cytometry tubes, washed once with PBS and centrifuged at 1500 rpm for 5 min. After removing the supernatant, each sample was re-suspended by gentle vortex in 100 μL LIVE/DEAD Fixable Near-IR Dead Cell Stain (1 μL stock in 1 mL PBS; Life Technologies) to stain for dead cells. Samples were incubated in the dark on ice for 20 min., and then washed with PBS and the supernatant discarded. Fluorescent cell surface staining antibodies were added to samples to a total of 100 μL per sample in PBS. Following incubation on ice for 30 min., samples were washed again and the resulting pellet re-suspended in 200 μL PBS for immediate flow cytometry analysis, or fixed for subsequent analysis at a later date. All washing and staining stages were carried out in the absence of light. For all studies cells were acquired on a Dako Cyan flow cytometer (Dako Cytomation), median fluorescent intensity (MFI) was measured for each cell surface marker and data were analysed using FlowJo software (Tree Star version 8.8.6). Data were also shown as representative flow cytometry plots. All antibodies were purchased from eBioscience/Thermofisher or BD Biosciences and expression quantified relative to the appropriate isotype control. A full list of antibodies, cytokines, volumes used and product numbers is shown in Supplemental Table 1. Rat IgG antibodies conjugated with matching fluorophores were used for the isotype control stain, and compensation colours were also added.

### 2.4 Microscope fluorescence imaging

For fluorescence microscopy imaging DCs were washed with PBS and stained with 100 nM MitoTracker Green FM (Invitrogen) diluted in RPMI 1640 medium without FBS at 37 °C for 25 min. Cells were washed and covered with mounting medium containing DAPI (VECTASHIELD) for nuclear staining. Images were taken using the Zeiss LSM780 confocal microscope. Two channels were selected: excitation at 490 nm – emission 416 for mitochondria stain based on MitoTracker Green FM and excitation at 360 nm and emission at 460 nm for nuclei stained with DAPI.

### 2.5 Quantitative real-time PCR

Total RNA was extracted by phenol/chloroform method after cell lysis in TRIzol (Life Technologies/Invitrogen). 0.3−0.5 μg RNA was reverse transcribed with random hexamers using TaqMan reverse transcription reagents (Thermofisher/Applied Biosystems). Quantitative real-time PCR for 18S rRNA, *VDR*, *CYP24A1* and *CYP27B1* was then performed on an Applied Biosystems 7900 machine using assays on demand from Applied Biosystems: 18S rRNA, (4319413E); *VDR* (Hs00118624 CE); *CYP24A1* (Hs00167999_m1); *CYP27B1* (Hs01096154_m1). Amplification of cDNAs involved incubation at 50 °C for 2 min. and 95 °C for 10 min. followed by 40 cycles of 95 °C for 15 s and 60 °C for 1 min. mRNA expression was statistically analysed as raw delta Ct values and fold-change in expression as originally described for quantification of PCR products [22].

### 2.6 Measurement of oxygen consumption

Measurement of mitochondrial oxygen consumption were performed based on previously reported studies [23,24]. Following the generation of different DC types using culture methods described above, the resulting DC populations were resuspended in serum-free RPMI at 37 °C and 3−5 × 10^5^ cell per assay and assessed for respiration using the following protocol. Samples were added to chambers in an Oroboros O2k-FluoRespirometer (Oroboros Inc., Innsbruck, Austria) and compounds used to induce different types of respiration were then sequentially added to these chambers via an integrated aperture in the FluoRespirometer, over a period of 12 min as follows: oligomycin at 2.5 μM (inhibitor of ATP synthase – measure ATP-coupled respiration); FCCP at 1 μM, until maximal respiration; rotenone at 0.5 μM (inhibitor of complex I - measures complex II linked respiration); antimycin A at 2.5 μM (inhibitor of complex III - stops respiration and measures residual oxygen consumption). Raw oxygen consumption rate changes were recorded over the 12 min period of analysis and processed to estimate different bioenergetic related parameters such as basal respiration, ATP-linked respiration and maximal capacity of ETC.

### 2.7 Mass spectrometry analysis of ^13^C-glucose and ^13^C-glutamine metabolism

To assess effects of 1,25D on glycolysis and the TCA cycle metabolic tracer analyses were carried out using uniformly-labeled ^13^C_6_-glucose or ^13^C_5_-glutamine as substrates as described previously [25]. Briefly, in the last 24 h of cell culture, conventional medium was replaced by DMEM supplemented with 10 % FBS, [U-13C] glutamine (2 mM) or glucose (10 mM). Following culture, cells were harvested and washed with NaCl (0.1 %). Metabolite extraction was performed by a gradient of 2.5 parts methanol: 2.5 parts of chloroform: 0.625 parts of H_2_O containing D-6-acid glutaric as internal reference. The extracted polar fraction was analysed to study metabolism in glycolysis and TCA cycle. For analysis of fatty acid synthesis, the extracted non-polar fraction was utilised. In the case of the latter, the replacement of RPMI media with DMEM media was carried out in day 3 of cell culture. In this case, a gradient of 50 % chloroform: 50 % methanol containing C17:0, heptadecylic acid (Sigma Aldrich) was used as an internal reference. The non-polar fraction was extracted and dried with nitrogen gas. Metabolites that differ in mass due to carbon composition are known as isotopologues and were represented as M + n; where n is the number of ^13^C atoms incorporated. Using mass spectrometry, metabolites and their isotopologues were quantified and their relative abundance was calculated. Metabolite abundance was normalized by the internal standard and total ion count. The incubation for labelled ^13^C-substrates was determined based on previous reports from our group in which a steady state equilibrium was achieved [25,26].

### 2.8 Statistical analysis

GraphPad Prism 7.0a software (GraphPad) was used for graphical summary and statistical analysis. FACS and ELISA data was statistically tested using one-way ANOVA with post-hoc Tukey’s test. For each test a 95 % confidence level was used. For Oxigraph data, raw data was processed to obtain basal respiration, maximal capacity and ATP linked respiration parameters. For each comparison, an n = 4 separate donor monocyte preparations were used for each treatment. To find significant differences in these respiration parameters a paired student’s *t* test was applied for each parameter comparing the 1,25D treated (itolDC cells) to untreated (iDC). For mass spectrometry analysis of metabolism relative abundance of isotopologues of different metabolites was measured. These isotopologues differ in the number of “labelled” carbons represented as M + n, where n is the number of carbons. For each experiment (glucose, glutamine and palmitate labelled experiment) iDC and itolDC were obtained from 5 different donors. To report significance differences in the relative abundance of each isotopologue of a metabolite, multiple paired student’s *t* test were performed. Metabolite tracer pool and glucose aportation: To compare differences in the overall metabolite pool and glucose usage to form polar metabolites between iDC and itolDC a paired student’s *t* test was used.

## 3 Results

### 3.1 1,25D regulates DC phenotype in the presence and absence of immunogenic stimulus

Differentiation of human peripheral blood monocytes to DC using 1,25D in the presence or absence of LPS was associated with specific changes in DC phenotype (Fig. 1). Relative to vehicle-treated iDC, LPS-induced mDC showed increased numbers of HLA-DR^+^ cells, and increased MFI for CD11c, CD80, CD83, CD86, CD209 and HLA-DR (Fig. 1B and C), consistent with a mature antigen-presenting phenotype [27,28]. Relative to mDC, mtolDC demonstrated decreased HLA-DR positivity, and suppressed CD80, CD83, CD86, CD209 and HLA-DR MFI (Fig. 1B and C), consistent with a tolerogenic DC phenotype [29,30]. The tolerogenic nature of 1,25D-induced itolDC is also illustrated by comparison with vehicle-treated iDC, with decreased numbers of CD80^+^ and HLA-DR^+^ cells, and CD14^+^ cells in itolDC. The MFI for CD80, CD86 and HLA-DR was also decreased in itolDC relative to iDC, whilst CD14 MFI was increased (Fig. 1B and C). Further characterisation of the DC sub-types was carried out by analysing expression of mRNAs for different components of the vitamin D signalling and metabolic system (Fig. 1D). *VDR* was expressed by all DC types, but was increased in LPS-treated DC (2.3- and 3.4-fold respectively in mDC and mtolDC). Both itolDC and mtolDC exhibited sensitivity to 1,25D as demonstrated by increased expression of the vitamin D catabolic enzyme *CYP24A1* (7.4- and 20.2-fold respectively in itolDC and mtolDC). By contrast, treatment with LPS alone (mDC) suppressed *CYP24A1.* LPS treatment also induced expression of the vitamin D activating enzyme *CYP27B1* (75.5- and 55.7-fold respectively in mDC and mtolDC), but treatment with 1,25D alone had no effect on *CYP27B1*. Subsequent data analyses were colour-coded according to phenotype colours shown in Fig. 1A.

### 3.2 1,25D-induced itolDC are characterised by increased oxidative phosphorylation

**Fig. 2:**
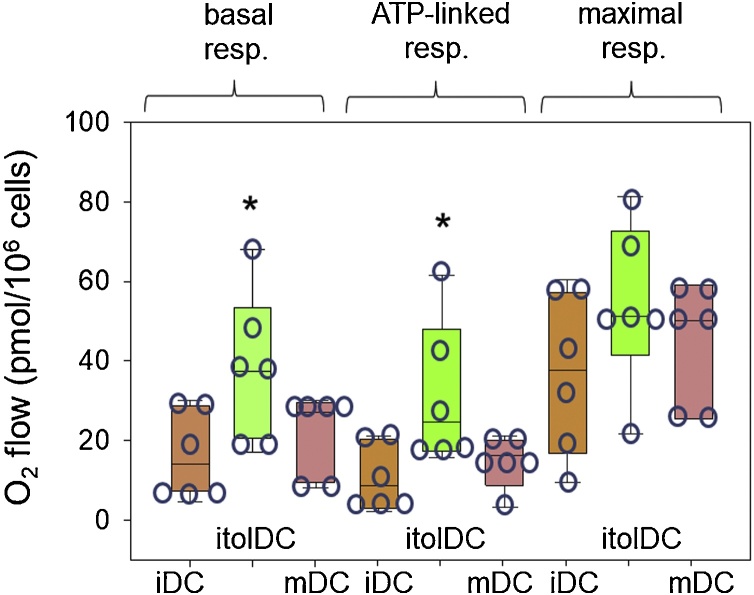
**1,25D-induced oxidative phosphorylation in itolDC**. Oxygen consumption rate (OCR) in IDC, itolDC and mDC. DC were differentiated for 6 days as described in Fig. 1A and then incubated in an Oroboros O2k-FluoRespirometer with integrated injection of specific factors to represent conditions of: i) basal respiration; ii) oligomycin-induced inhibition of ATP production; iii) FCCP uncoupling of electron transport chain; iv) antimycin A/rotenone inhibition of electron transport over a 12 min period. Quantification of replicate donor experiments for basal respiration, ATP-linked respiration and maximal respiration capacity data was carried out for multiple time points with each cell type. Data are the individual replicate values median and upper/lower quartiles for n = 6 replicate donor analyses. * = statistically different from iDC values, *p* < 0.05.

Oxygen consumption rates (OCR) were measured in different populations of DC to assess the effects of 1,25D and LPS on metabolic remodelling in the different DC phenotypes (Fig. 2). Relative to iDC, 1,25D-induced itolDC showed increased basal respiration and ATP-linked respiration. By contrast, induction of mDC via LPS-treatment had no significant effect on basal, ATP-linked or maximal respiration (Fig. 2). The effects of 1,25D on OCR were associated with 6-day treatment, as iDC or treated with 1,25D for 24 h showed no significant changes in OCR (data not shown).

### 3.3 1,25D-induced itolDC show increased TCA cycle metabolism

**Fig. 3:**
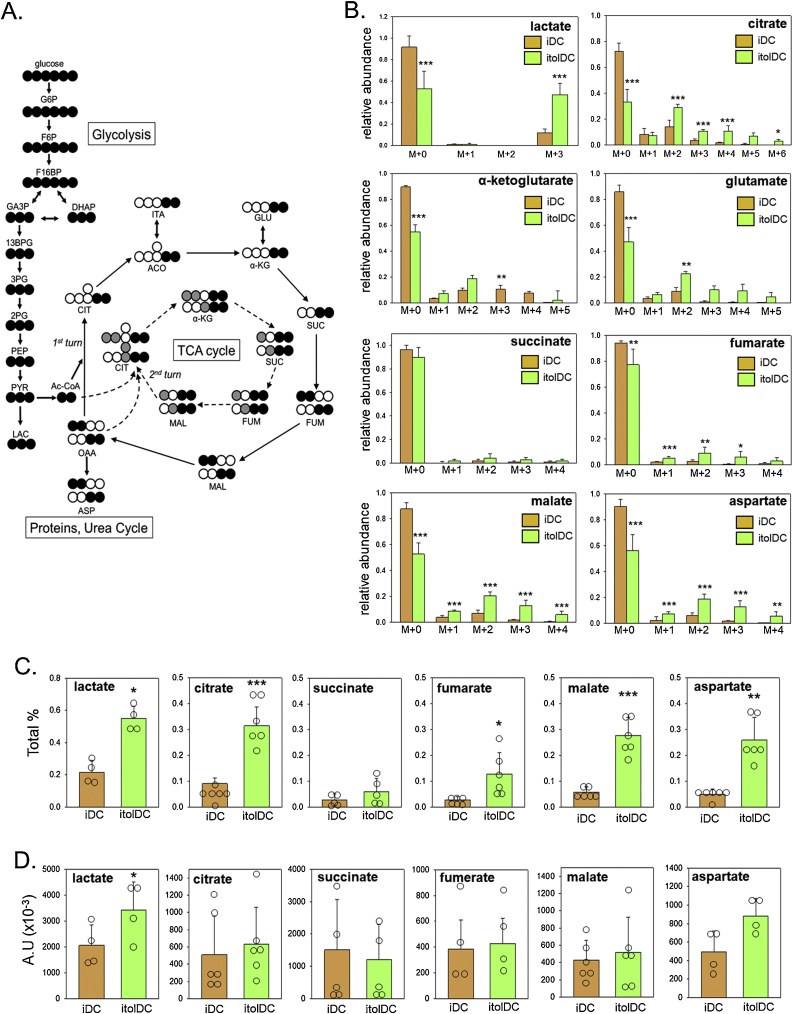
**Tracer metabolite analysis of glucose metabolism in iDC and itolDC**. A. Schematic representation for incorporation of 13C-labeled glucose in polar TCA cycle metabolites through glycolysis and TCA cycle metabolism. B. Relative abundance of unlabelled (M + 0) and 13C-labeled (M + 1, M + 2, M + 3, M + 4, M + 5, M + 6) TCA cycle metabolites in iDC and itolDC. C Total % of TCA cycle metabolites in iDC vs itolDC produced due to glucose consumption. D. Total pool of TCA cycle metabolites in iDC vs itolDC (arbitrary units A.U.). Abbreviations: glucose 6-phosphate (G6P); fructose 6-phosphate (F6P); fructose 1,6-bisphosphate (F16BP); glyceraldehyde 3-phosphate (GA3P); dihydroacetone phosphate (DHAP); 1,3-bisphosphoglyceric acid (13BPG); 3-phosphoglyceric acid (3 PG); 2-phosphoglyceric acid (3 PG); phosphoenolpyruvic acid (PEP); pyruvate (PYR); lactate (LAC); acetyl-CoA (Ac-CoA); citrate (CIT); aconitate (ACO); itaconate (ITA); α-ketoglutarate (α-KG); glutamate (GLU); succinate (SUC); fumarate (FUM); malate (MAL); oxalo-acetate (OAA); aspartate (ASP). Data for Total % of TCA cycle metabolites and Total pool of TCA cycle metabolites are shown as the individual replicate values and mean ± SD for n = 5 separate donor monocyte preparations. Data for relative abundance of TCA metabolites are shown as mean ± SD for n = 5 separate donor monocyte preparations. * = itolDC values statistically different from iDC, *p* < 0.05, ** *p* < 0.01, *** *p* < 0.001.

Data in Fig. 1, Fig. 2 indicate that DCs treated with 1,25D alone show an enhanced tolerogenic phenotype (itolDC) which was associated with increased mitochondrial respiration. Further studies were therefore carried out to more precisely define the metabolic remodelling induced by 1,25D in itolDC. DCs treated with fully labelled U-^13^C_6_-glucose for the final 24 h of culture were analysed by mass spectrometry to quantify incorporation of the isotope into the resulting polar (hydrophilic) tracer metabolites (Fig. 3). The pathway for incorporation of ^13^C_6_-glucose into metabolites via glycolysis and the TCA cycle is shown in Fig. 3A. Following glycolysis, the carbons from ^13^C_2_-acetyl-CoA are committed to the TCA cycle through citrate synthase, and remain in every metabolite generated in the first round of the cycle, reflected in the relative abundance of m + 2 isotopomers (Fig. 3B). Further incorporation of ^13^C-acetyl-CoA in a second round of the TCA cycle generates m + 4 and m + 6 isotopomers of citrate which were also increased in itolDC. M + 3 and mM + 5 isotopomers reflecting consecutive rounds of TCA cycle and pyruvate carboxylase activity were also enhanced in itolDC (Fig. 3B).

Consistent with the 1,25D-induced glycolysis and TCA cycle shown in Fig. 3B, itolDC also showed higher incorporation of glucose carbons into citrate (66.91 %), aspartate (43.72 %), malate (47.37 %), fumarate (22.71 %) and lactate (47.09 %) relative to vehicle-treated iDC (27.69 %, 9.79 %, 12.53 %, 5.9 % and 8.56 % respectively), whilst incorporation into succinate showed no significant change (Fig. 3C). Despite the changes in ^13^C-glucose incorporation outlined above, the total amount of each metabolite measured by mass spectrometry showed that only lactate showed significantly increased steady state levels in itolDC compared to untreated iDC (Fig. 3D).

**Fig. 4:**
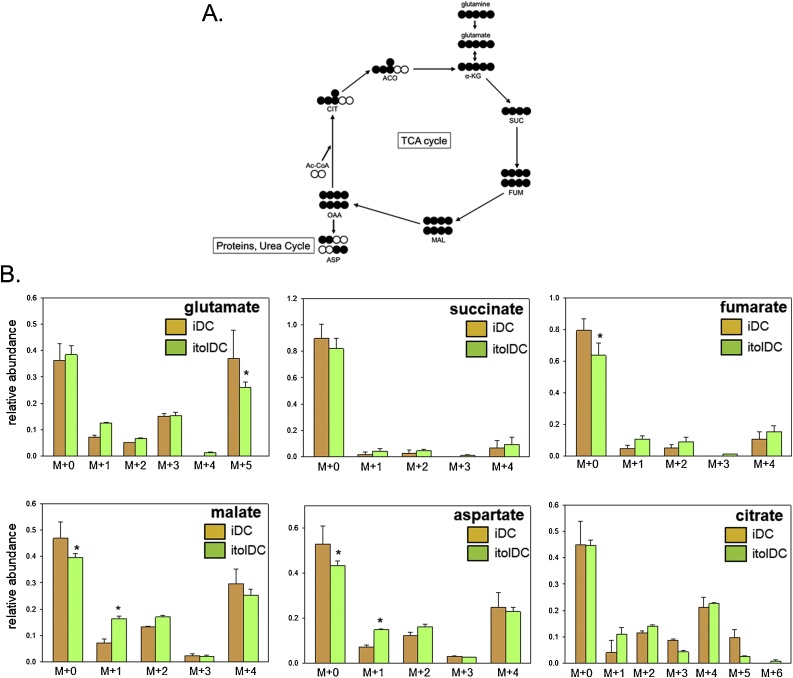
**Tracer metabolite analysis of glutamine metabolism in iDC and itolDC**. A. Schematic representation showing incorporation of 13C-labeled glutamine in polar TCA cycle metabolites as a consequence of TCA cycle metabolism. B. Relative abundance of unlabelled (M + 0) and 13C-labeled (M + 1, M + 2, M + 3, M + 4, M + 5, M + 6) TCA cycle metabolites in iDC and itolDC. Abbreviations: acetyl-CoA (Ac-CoA); citrate (CIT); aconitate (ACO); α-ketoglutarate (α-KG); succinate (SUC); fumarate (FUM); malate (MAL); oxalo-acetate (OAA); aspartate (ASP). Data are shown as mean ± SD for n = 5 separate donor monocyte preparations. * = itolDC values statistically different from iDC, p < 0.05.

To determine the wider metabolic implications of increased utilisation of glucose carbons by itolDC, further tracer metabolite analyses were carried out using ^13^C- glutamine as a substrate (Fig. 4A). In Fig. 3, there was incorporation of ^13^C-glucose into multiple isotopologues for each TCA metabolite (excluding succinate) in 1,25D-induced itolDC. By contrast, ^13^C-glutamine was only significantly incorporated into the m + 1 isotologue for malate and aspartate, and the m + 5 isotopologue for glutamate in itolDC (Fig. 4B). These data suggest that DC TCA responses to 1,25D predominantly involve metabolism of glucose.

### 3.4 1,25D induces fatty acid synthesis in itolDC

**Fig. 5:**
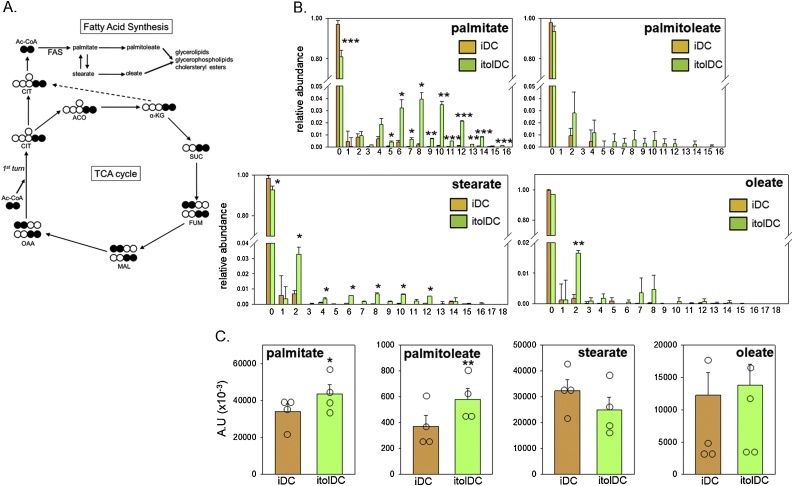
**Tracer metabolite analysis of glucose metabolism to fatty acid precursors in iDC and itolDC**. A. Schematic representation showing incorporation of 13C-labeled glucose in non-polar (lipophilic) TCA cycle metabolites as a consequence of glycolysis and TCA cycle metabolism. B. Relative abundance of unlabelled carbon (M + 0) (0) and 13C-labeled isotopologue (M + 1 – M + 18) [[1], [2], [3], [4], [5], [6], [7], [8], [9], [10], [11], [12], [13], [14], [15], [16], [17], [18]] in iDC and itolDC. C. Total pool of stearate, oleate, palmitoleate, and palmitate in iDC vs itolDC (arbitrary units, A.U.). Abbreviations: acetyl-CoA (Ac-CoA); citrate (CIT); aconitate (ACO); α-ketoglutarate (α-KG); succinate (SUC); fumarate (FUM); malate (MAL); oxalo-acetate (OAA). Data for the Total pool of stearate, oleate, palmitoleate, and palmitate are individual replicate values and mean ± SD for n = 5 separate donor monocyte preparations. Data for relative abundance of TCA metabolites are shown as mean ± SD for n = 5 separate donor monocyte preparations. * = itolDC values statistically different from iDC, *p* < 0.05, ** *p* < 0.01, *** *p* < 0.001.

Data in Fig. 3, Fig. 4 show the effect of 1,25D on polar, hydrophilic metabolites derived from ^13^C_6_-glucose and ^13^C_5_-glutamine. To assess possible effects of 1,25D on hydrophobic metabolites, further analysis of the non-polar fraction of cell extracts after incubation with ^13^C-glucose was carried out (Fig. 5). The pathway from TCA cycle (citrate) to fatty acids is shown in Fig. 5A. The relative abundance of different fatty acid isotopomers (Fig. 5B) showed significant incorporation of ^13^C-glucose for only palmitate. However, analysis of the total pool of different fatty acids in iDC and itolDC (Fig. 5C) showed that both palmitate and palmitoleate were increased significantly in 1,25D-induced itolDC. Collectively these data indicate that induction of itolDC by 1,25D is associated with a significant increase in *de novo* fatty acid synthesis via glucose and citrate.

### 3.5 Inhibition of fatty acid synthesis suppresses itolDC induction by 1,25D

**Fig. 6:**
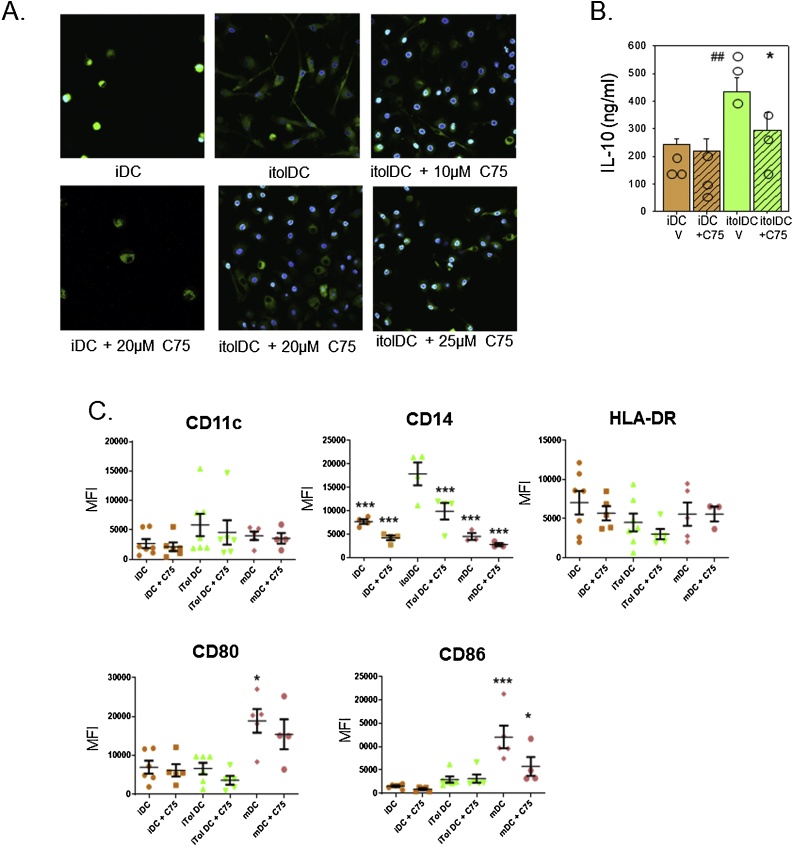
**Inhibition of fatty acid synthesis suppresses effects of 1,25D on CD14 and IL-10 in itolDC.** A. Mitotracker and DAPI immunofluorescence in iDC and 1,25D-induced itolDC treated with vehicle or the fatty acid synthase inhibitor C75 (10-25 μM). B Concentration of IL-10 in supernatants from iDC and 1,25D-induced itolDC cultured in the absence (vehicle, V) or presence of C75 (20 μM). C flow cytometric analysis of CD14, HLA-DR, CD80 and CD86 in iDC and 1,25D-induced itolDC cultured in the absence (vehicle, V) or presence of C75 (20 μM). Data are shown as frequency of expression for analysis of n = 4-5 separate donor PBMC preparations. * = statistically different from itolDC, *p* < 0.05, ** *p* < 0.01, *** *p* < 0.001.

The functional significance of fatty acid synthesis in 1,25D-induced itolDC was assessed using the fatty acid synthase inhibitor C75 [31]. Immunofluorescence showed that, relative to the more rounded morphology of vehicle-treated iDC, treatment with 1,25D produced a pronounced dendritic morphology in itolDC. This effect was abrogated in itolDC treated with C75 which exhibited fewer dendrites and a more rounded morphology (Fig. 6A). Analysis of DC culture supernatants also showed that inhibition of fatty acid synthase by C75 suppressed IL-10 production by itolDC (Fig. 6B). Flow cytometry showed that C75 had no effect on HLA-DR, CD80 or CD86 expression by iDC, itolDC or mDC, but was associated with significant suppression of 1,25D-induced CD14 expression by itolDC (Fig. 6B).

## 4 Discussion

Regulation of cellular metabolism plays a critical role in immune function, promoting changes in energy production and biosynthesis that underpin key innate immune responses to infection and tissue damage, while providing the metabolic basis for expansion of lymphocyte function in adaptive immune responses [32]. Antigen presenting cells such as macrophages and DC are particularly dependent on the regulation of metabolic pathways to maintain phenotype plasticity, whilst integrating antibacterial responses and T cell activation with tolerogenic attenuation of immune function to limit tissue damage [33,34]. Induction of glucose consumption and glycolysis plays a crucial role in the activation of DCs and their ability to acquire, process and present antigen [35]. Notably, inhibition of hexokinase, the first enzymatic step in glycolysis, potently blocks DC activation [35]. At least part of the glycolytic metabolic reprogramming associated with DC activation involves the production of lactate, with recent studies showing that lactate acts as a crucial signalling molecule for tolDC in the generation of regulatory T cells (Treg) [36]. However, DC tolerogenicity is also strongly influenced by lipid metabolism, with elevated glycolysis and electron transport being linked to increased fatty acid oxidation (FAO) in tolDC [37]. Previous studies by ourselves and others have shown that the tolerogenic effect of 1,25D on DCs is fundamentally associated with metabolic reprogramming in the form of increased glycolysis, oxidative phosphorylation and TCA cycle activity [20,21]. In data presented here we show that treatment of DC with 1,25D alone is sufficient to generate itolDC that exhibit significant metabolic reprogramming independent of immune activation, with enhanced fatty acid synthesis playing a central role in the generation of the itolDC phenotype.

The itolDC in the current study are phenotypically similar to previously reported tolDC generated using combined treatment with 1,25D (100 nM) and dexamethasone (DEX) [37], with decreased expression of CD86 and CD80, increased expression of CD14, and relatively little change in CD11c relative to mDC. 1,25D/DEX-tolDC showed similar glycolytic activity to LPS-induced mDC but glycolytic reserve, respiratory capacity and metabolic plasticity were significantly higher in tolDC [37]. Increased glycolysis and oxidative phosphorylation have also been described for tolDC induced by 1,25D in the absence of DEX. However, in this case 6 day treatment with 1,25D to generate tolDC was accompanied by activation with LPS for the final 24 h, thus generating an mtolDC phenotype [20]. Nevertheless, this study showed that the metabolic reprogramming induced by 1,25D in combination with LPS was distinct from that observed for other tolerogenic treatments such as DEX + LPS [20]. Specifically, 1,25D was shown to promote metabolic reprogramming via activation of PI3kinase-Akt-mTOR pathways, with inhibition of these pathways acting to inhibit the tolerogenic effects of 1,25D on DC. By contrast, the effects of other promoters of DC tolerogenesis such as DEX and IL-10 were unaffected by manipulation of the PI3kinase-Akt-mTOR pathway [20]. These observations, in combination with the data present in the current study, indicate that 1,25D alone is sufficient to promote the metabolic reprogramming required for a tolerogenic DC phenotype.

In addition to enhanced aerobic glycolysis and lactate production, 1,25D + LPS-induced mtolDC have also been shown to exhibit increased TCA cycle activity. Tracer metabolite analysis using ^13^C-glucose has shown that 1,25D promotes 13C-labeling of several TCA metabolites, consistent with increased incorporation of glucose into the TCA cycle [38]. In the current study we observed a similar significant increase in TCA cycle flux in itolDC induced by 1,25D alone. Importantly, by using both ^13^C-glucose and ^13^C-glutamine as TCA cycle substrates, we were able to confirm that the metabolic reprogramming actions of 1,25D are focused primarily on strongly enhanced incorporation of glucose into lactate and the TCA cycle (Fig. 3B and C). Nevertheless, in general the overall concentrations of individual TCA metabolites were not significantly altered in itolDC (Fig. 3D). This suggests that these metabolites are highly incorporated into other metabolic pathways in DC treated with 1,25D, and this was the primary rationale for carrying out further studies of lipophilic metabolites.

Both glycolysis and the TCA cycle can contribute to further metabolic pathways such as the proteins and lipid synthesis. In the current study we have shown for the first time that metabolic reprogramming effects of 1,25D on glucose consumption, glycolysis and TCA cycle activity in DC are associated with increased generation of fatty acids. Fatty acid synthesis appears to play a pivotal role in the generation and survival of DCs, with inhibition of FAS decreasing DC development but also enhancing T cell activation by DC, with these effects being mediated partly through increased ER stress [39]. In a similar fashion, exogenously added polyunsaturated fatty acids have been shown to block T cell activation by DC [40]. It is therefore possible that the increased synthesis of fatty acids by 1,25D itolDC may act in a paracrine fashion to affect T cell responses. This need not necessarily be restricted to paracrine actions on T cells, as previous studies have described potent effects of palmitic acid on DC function, with the fatty acid acting as a toll-like receptor 4 ligand [41]. Thus, synthesis of fatty acids may provide a mechanism for intra-DC signalling.

It is important to recognise the effects of fatty acids on DC function may be dependent on the context of DC development, as hepatic DC with low levels of fatty acids showed a tolerogenic phenotype and promoted Treg generation [42]. Palmitate and palimoleate are fatty acid precursors but they may also themselves exert anti-inflammatory effects, with palmitoleate functioning as a lipokine to regulate AMP kinase [43], and suppress inflammation [44]. Collectively these observations indicate that the generation of palmitate and palmitoleate from glucose may be a pivotal component of the metabolic function of 1,25D in DC. It was therefore interesting to note that although inhibition of FAS in itolDC resulted in profound changes in DC morphology, the cells remained viable and exhibited relatively modest changes in expression of cell surface antigens. Consistent with the itolDC IL-10 analyses presented here, previous studies of inflammatory macrophages have shown that inhibition of FAS significantly suppresses expression of several cytokine markers, including IL-10 [45]. Given the fundamental importance of IL-10 for DC tolerogenesis [46] and the fact that our results indicate that FAS may play a key role in the ability of 1,25D to promote IL-10 production in tolerogenic DC, we suggest that fatty acid synthesis be a key metabolic pathway in the development of a tolerogenic phenotype in DC but further work is needed to understand the molecular links between the FAS pathway and DC phenotype. The role of CD14 in this process is less clear and the suppression of itolDC CD14 following FAS inhibition may simply reflect the elevated levels of CD14 in these cells. However, it is interesting to note that a subset of IL-10-secreting tolerogenic DC referred to as DC-10 have been described *in vivo* and *in vitro*, that express also express CD14 in contrast to iDC or mDC [47].

## 5 Conclusions

Data presented in this study show that 1,25D alone is sufficient to metabolically reprogramme DC, with increased glycolysis and lactate generation, and enhanced TCA cycling leading to fatty acid synthesis. Inhibition of fatty acid synthesis was sufficient to alter the morphological and IL-10 induction effects of 1,25D, indicating that this component of metabolism is crucial for DC tolerogenesis. However, it is likely that 1,25D coordinates a spectrum of changes in DC metabolism, notably including the potent generation of lactate in itolDC. Recent studies have shown the lactate itself is a pivotal DC-metabolite that promotes the generation of Treg [36], so that the induction of itolDC by 1,25D may involve altered DC morphology and function via enhanced fatty acid synthesis, as well as indirect effects on T cells via lactate generation. Further studies are also required to assess the *in vivo* significance of these observations. Previous reports have shown that DC exhibit significant capacity for localised synthesis of 1,25D from precursor 25D [9], so that it is possible that the variations in circulating 25D that conventionally define human vitamin D ‘status’ may exert concomitant effects on the metabolic profile of DC. The impact of this on immune function in vivo will be an important facet of future studies of vitamin D supplementation.

## Declaration of Competing Interest

The authors declare that there is no conflict of interest that could be perceived as prejudicing the impartiality of the research reported AMG was supported by a PhD Studentship from the Maastricht-Birmingham PhD Programme. DL was supported by a PhD studentship from the 10.13039/501100007155Medical Research Council. EB was supported by a MIDAS PhD studentship from the 10.13039/100010269Wellcome Trust. MH is supported by a Royal Society Wolfson Merit Award (WM130118) and 10.13039/100000002National Institutes of Health (AR063910).
