# Supplementary material for: Tolerogenic effects of 1,25-dihydroxyvitamin D on dendritic cells involve induction of fatty acid synthesis
Source: J Steroid Biochem Mol Biol. 2021 Jul;211:105891. doi: 10.1016/j.jsbmb.2021.105891 (PMC8223499; doi:10.1016/j.jsbmb.2021.105891)
Supplement: Supplementary file 1 [file mmc1.docx]

| **Antigen** | **Fluorophore** | **Clone** | **Isotype** | **Company** | **Catalogue Number** |
| --- | --- | --- | --- | --- | --- |
| CD11c | PeCy7 | B-LY6 | mouse BALB/c IgG1,k | BD Biosciences | 561356 |
| CD14 | PerCP | MΦP9 | Mouse BALB/c IgG2b, κ | BD Biosciences | 340585 |
| CD40 | APC | 5C3 | Mouse IgG1, κ | BD Biosciences | 555591 |
| CD80 | PE | L307 | Mouse C3H | BD Biosciences | 557227 |
| CD80 | APC | 2D10 | Mouse IgG1κ | Miltenyi Biotec | 130-097-204 |
| CD83 | PE | HB15e | Mouse IgG1, κ | BD Biosciences | 556855 |
| CD86 | FITC | 2331 (FUN-1) | Mouse BALB/c IgG1, κ | BD Biosciences | 555657 |
| CD86 | BV421 | 2331 (FUN-1) | Mouse BALB/c IgG1, κ | BD Biosciences | 562432 |
| CD209 | APC | DCN46 | Mouse IgG2b, κ | BD Biosciences | 551545 |
| HLA-DR | FITC | G46-6 | Mouse IgG2a, κ | BD Biosciences | 555811 |
| HLA-DR | APC | G46-6 | Mouse IgG2a, κ | BD Biosciences | 559866 |

**Supplemental Table 1**
